# Reductions in HIV incidence are likely to increase the importance of key population programmes for HIV control in sub‐Saharan Africa

**DOI:** 10.1002/jia2.25727

**Published:** 2021-06-30

**Authors:** Geoff P Garnett

**Affiliations:** ^1^ Tuberculosis and HIV Strategic Team Bill & Melinda Gates Foundation Seattle WA USA

**Keywords:** HIV epidemiology, key populations, cost‐effectiveness, transmission dynamics, reproductive numbers, epidemic control

## Abstract

**Introduction:**

An efficient HIV response requires that resources be focussed on effective interventions for those most at risk of acquiring and transmitting infection. As HIV epidemics evolve the distribution of HIV across key and other populations will change. Here, the epidemiological concepts underpinning these changes are described and the importance of appropriate allocation of effective interventions is discussed.

**Discussion:**

In many sub‐Saharan African countries HIV epidemics have been categorized as “generalized,” and HIV testing, treatment and prevention interventions have focussed on the “general” population. As HIV epidemics are better controlled the relative importance of “key” populations will increase, dominating the ongoing burden of disease and providing the potential for repeated outbreaks of HIV if interventions are relaxed. The basic reproductive number (R_0_) describes the potential for an infectious disease to spread at the boundary of invasion or elimination, whereas the effective reproduction number (R_t_) describes the current potential for spread. Heterogeneity in risk means that while R_t_ is temporarily below one and prevalence declining, the R_0_ can remain above one, preventing eventual elimination. Patterns of HIV acquisition are often used to guide interventions but inadequately capture the transmission dynamics of the virus and the most efficient approach to controlling HIV. Risks for HIV acquisition are not identical to risks for HIV transmission and will change depending on the epidemiological context. In addition to the challenges in measuring HIV transmission dynamics, there is a tension between using epidemiology to drive the HIV response and the social and political realities constraining how programmes and providers can practically and appropriately focus on key populations and maintain political support. In addition to being well focussed, interventions need to be effective and cost‐effective, which requires a better understanding of packages of interventions rather than specific tools.

**Conclusions:**

Continued control of HIV will increasingly rely on resources, programmes and interventions supporting key populations. Current epidemiological and programmatic approaches for key populations in sub‐Saharan Africa are insufficient with a need for an improved understanding of local epidemiology and the effectiveness of interventions.

## 1 INTRODUCTION

The salience of “key populations” in HIV epidemics in sub‐Saharan Africa has shifted over time. Over the first decade of the AIDS pandemic it became clear that HIV could be heterosexually transmitted and that the virus was particularly widespread in the “general population” in some regions of sub‐Saharan Africa [1, 2]. The categorization of HIV epidemics as nascent, concentrated or generalized was introduced to help design surveillance using serological surveys from antenatal clinic attendees in generalized epidemics, and purposive samples of identifiable “high‐risk groups” in concentrated epidemics [3]. Unfortunately, categorizing epidemics as generalized has allowed neglect of the key populations that are particularly affected by HIV: men who have sex with men (MSM), transgender women, people who inject drugs (PWID), sex workers and prisoners [4]. In the “generalized epidemics” of Southern and Eastern Africa substantial resources have been directed in an undifferentiated manner with mass testing and treatment and much less attention has been paid to epidemics among key populations, particularly MSM and transgender women [5].

Throughout the course of the HIV pandemic there have been barriers to acknowledging epidemiological insights about patterns of HIV acquisition and transmission and providing appropriate support for key populations. Blame, stigma, discrimination and criminalization threaten the rights of key populations and political calculus often directs resources to those with less risk [6]. Key populations have likely been disproportionately affected and at risk of HIV, but there has been a failure in much of sub‐Saharan Africa to understand their size, the levels of risk of both acquisition and transmission, and how best to intervene for their benefit. An effective response to HIV, minimizing the morbidity and mortality across populations, depends upon an understanding of HIV epidemiology and effective interventions, and programming of resources to follow that evidence. Effective coverage of key populations also requires that interventions meet the populations’ needs, and that they avoid discrimination and intolerance [7]. With overall reductions in HIV incidence the need to address the problems experienced by key populations only increases.

Initial declines in HIV incidence in some countries of Southern and Eastern Africa occurred around the year 2000, before the scale‐up of antiviral treatment, and were associated with reductions in numbers of sex partners and increased condom use [8, 9, 10]. More recently, voluntary medical male circumcision (VMMC), and antiviral treatment have further reduced HIV incidence [11, 12]. In Southern and Eastern Africa new HIV infections per year have declined by 38% over the last decade, and in Western and Central Africa they have declined by 25% [13]. These recent declines have been achieved mainly through the scale‐up of antiviral treatment, which on aggregate by 2019 had reached 78% of adult women and 65% of adult men living with HIV in Southern and Eastern Africa, and 67% and 49%, respectively, in Western and Central Africa [13]. However, aggregate data likely mask a failure to treat some subpopulations, which may explain the mismatch between predicted and observed reductions in HIV [14]. In a recent systematic review of data on HIV treatment cascades there were insufficient data regarding treatment coverage in key populations [15]. Unfortunately, as HIV incidence declines in the “general” population it is likely that the relative risk experienced by key populations compared to other group will increase, since HIV will concentrate in “core” groups [16], In addition, there may be perverse consequences of success in reducing the HIV burden, since fewer resources may be allocated when the general trends are perceived to be going in the right direction. It is important that resources and interventions are appropriately focussed on key populations in the region.

## 2 DISCUSSION

Infectious diseases, like HIV, spread through a network of contacts with a likelihood of transmission and acquisition and the extent of that spread depends upon the characteristics of the network of contacts, the course of infection and biology of transmission. Contacts are embedded in a social, cultural and political context. The interaction of these factors determines the basic reproductive number of infection (R_0_), which is the *average* number of new infections generated by an infection in an entirely susceptible population (Table 1) [17]. Only if R_0_ is greater than one is a sustained epidemic possible. Once an epidemic takes off, some contacts will already be infected and the basic reproductive number is reduced to an effective reproductive number (R_t_) [17]. Eventually, the number of infections will saturate in a population so that with each new infection replicating itself, an endemic steady state is reached. Changes in patterns of contact or in the biology of the infection could change the R_0_, while interventions reducing infectiousness or risky contacts reduce the R_t_ [18]. Interventions can reduce the R_t_ below one, driving down incidence and, in an idealized “homogenous population,” eventually eliminate infection.

**Table 1:** Key terms and concepts in infectious disease transmission dynamics and how they are likely to change as HIV epidemics progress in sub‐Saharan Africa

	Definition	Significant value	Expected change as HIV is better controlled
Basic Reproductive number (R_0_)	The average number of new infections generated by one infection in an entirely susceptible population	Above one infection can lead to epidemics	If it remains below one epidemics will be eliminated, but despite current declines in infection, it may still be above one especially in “key” populations and a new steady‐state incidence could emerge
Effective Reproductive Number (R_t_)	The current average number of new infections generated by one infection^a^	One in the endemic steady state	Currently less that one with prevalence declining. Is likely to increase again to one and a new endemic steady state emerge
Incidence/mortality ratio	The net change in infection numbers	If less than one prevalence is declining	The lower the incidence/mortality ratio the faster numbers of people living with HIV will decline
Core group	That part of the population where someone infected with HIV will on average transmit to more than one other person		Necessary for epidemics to be sustained. As average risk declines (e.g. antiviral reduces transmission likelihood) then the core group will reduce in size and involve more “risky” behaviour
Risk factor	A variable that is associated either causally or through correlation with a change in the chances of acquiring HIV		As HIV prevalence declines the chances of HIV acquisition associated with a given level of a risk factor will decline. The relative risk is likely to increase in relation to a none “exposed” group where chances of acquisition also decline
Population attributable fraction (PAF)	The proportion of infections that would not occur if a risk factor is not present		For HIV one needs to account for transmission as well as acquisition so transmission population attributable fractions (tPAFs) over time can be modelled by comparing HIV epidemics with and without a risk factor. As HIV is controlled remaining risk factors will have a higher tPAF

^a^ It is ambiguous as to whether this is an average of extant infections over their past and future course or infections starting at the given time point.

Populations, though, are not homogeneous, and this is particularly true for HIV and other sexually transmitted infections, where patterns of contact and likelihood of transmission can vary greatly. Heterogeneity in the pattern of contacts and the likelihood of transmission between contacts can dramatically alter the potential for epidemic spread, the eventual scale of an epidemic and the impact of interventions [19]. Some of the heterogeneity relevant to HIV is captured in the concept of “key populations.” If some have a greater risk of acquiring and transmitting infection, R_0_ is increased, making it easier for an epidemic to take off, but limiting the prevalence at which the epidemic will saturate as R_t_ rapidly falls [17]. Those with a greater likelihood of transmission have been described as a core group [20]. The network of contacts can be described by a pattern of mixing: if those with a higher likelihoods of transmission mix regularly with those of lower likelihoods the infection can spread further, whereas if mixing is more constrained then the infection will likewise be constrained to those with a higher risk [21, 22]. If interventions further reduce R_t_ and prevalence declines, infections will increasingly concentrate among those with the greatest likelihood or acquisition and transmission, where chains of transmission can be maintained. R_t_ will eventually rise back to one with a new, lower steady‐state incidence and prevalence. An R_t_ of one is the situation where the incidence of new HIV infections equals deaths from HIV, which has been defined by some as a threshold for “epidemic control” [23]. However, this threshold does not imply that prevalence will continue to fall indefinitely unless the R_0_ is also less than one, nor does it imply that continued interventions are unnecessary.

In Southern and Eastern Africa, HIV spread widely through the community reaching levels in some districts where half of young adults were living with HIV [8]. In Central and Western Africa, HIV spread was more limited with a greater fraction of infections being among sex workers and their clients [24]. Across Africa, the importance of key populations to HIV was underestimated by models looking at only risks for the heterosexual acquisition of HIV, or short‐term patterns of transmission [25, 26, 27]. From its peak, HIV incidence has declined through a mixture of natural epidemic dynamics (through saturation of the network of contacts and differential mortality of those with the greatest risk of acquiring and transmitting infection), sexual behaviour modification in response to HIV, and the improved coverage of interventions, particularly antiretroviral treatment [8, 10, 28].

The decline in HIV incidence likely means that R_t_ is below one. If R_t_ is less than one HIV is only headed towards elimination if R_0_ is also less than one. This may happen if interventions are maintained and continue to suppress the reproduction of HIV cases, even as the infections become more concentrated, making elimination over many decades possible. However, other scenarios are more likely, with a resurgence of HIV epidemics if a prior decline in incidence leads to investments and efforts to intervene being scaled back, or populations taking fewer precautions against HIV transmission. However, it is also possible to focus investments and interventions on the populations most at risk, thereby efficiently preventing the resurgence of epidemics.

The underlying changes in the basic and effective reproduction numbers have consequences for policy. First, the fact that HIV prevalence is declining and the spread of HIV is controlled does not mean there can be a let up on interventions; second, when HIV transmission is concentrated in key populations it should be possible to maintain control of HIV epidemics through interventions among key populations and relax more defuse efforts; third, failure to intervene effectively among key populations will likely undermine any hope of eliminating HIV.

The mathematical observations described above are relevant for the current state of HIV control in sub‐Saharan Africa, but they have not been without controversy [20]. Long before the mathematical description of “core groups” for sexually transmitted infections [29] blame was placed on the marginalized populations of sex workers and campaigns criminalized and stigmatized these populations [30]. This stigmatization and discrimination continued with HIV, with those at most risk vilified, and others with more risk of acquisition than transmission (transfusion recipients and neonates) described as “innocent victims.” Messages were tailored to suggest everyone was at risk, which had the advantage of making HIV everyone’s concern, but the disadvantage of steering resources away from those most in need [6]. It is axiomatic that for HIV epidemics to grow R_0_ had to exceed one. Furthermore, if there is heterogeneity in contacts and likelihood of transmission then some people living with HIV (PLHIV) will transmit to more than one other person, and some will transmit to fewer than one, but to infer fault is misguided both morally and practically.

The variables that determine someone’s risk of acquiring and transmitting HIV are not synonymous with the variables representing someone’s cultural, social and psychological identity. Key populations and their members may be mischaracterized as at risk of HIV depending upon behavioural and epidemiological context. Additionally, they may misperceive their own risk. Heterogeneities in risk and in correlations between risk and perceptions of identity may compromise the ability of programmes to reach some segments of the population, sometimes including those most at risk. An important programmatic goal for HIV control in sub‐Saharan Africa should be to match interventions to practical social and cultural descriptors and locations that will allow those at most risk to be reached by effective interventions.

Risk in epidemiology is often measured as the relationship for the individual between genetic, behaviour and environmental factors and the incidence of disease. This approach, which works for non‐communicable disease assumes a stable relationship between risk factors and disease and focuses on individual risks. For infectious diseases, like HIV, the importance of risk factors depends on the spread of infection within the network of contacts of the individuals, which in turn depends upon the (different) risks for acquisition and for transmission. Measuring who acquires HIV and focusing solely on that to guide programmes, overemphasizes risks of acquisition and underestimates the importance of risks of transmission. With heterogeneity in risks, the majority of those acquiring infection are likely to have only moderate risk. Preventing infection among those with a higher risk of acquisition and transmission could eliminate infections in those with moderate risk. For example, although young women in sub‐Saharan Africa are more likely to acquire HIV, they are less likely to transmit the infection than the older men they have contact with [31]. Preventing these men from acquiring HIV would also prevent the young women from acquiring HIV. Likewise, preventing infections among key populations is in theory likely to disproportionately reduce HIV incidence among other groups in the population. However, due to a lack of data, there has been uncertainty about the role of key populations in the high prevalence epidemics of rural sub‐Saharan Africa [32]. The role of key populations in the spread of HIV to other groups could be clarified using sequencing data and phylogenetics.

There are two ways of identifying the importance of key populations in the ongoing spread of HIV, but both rely on good data on the numbers in the key populations and their risk behaviours either through surveys or contact tracing/partner notification. Such data along with representative samples of the virus and phylogenetic analysis can help reconstruct transmission pathways and understand the importance of key populations [32]. Alternatively, detailed models of the network of sexual contacts and the transmission patterns of the virus can reconstruct the observed trends in HIV and identify the importance of key populations [26]. Models so constructed can also predict the future spread of HIV and the impact of HIV interventions, both focused on key populations and more diffuse populations.

If HIV becomes concentrated among key populations, then to be efficient HIV programmes will need to focus on working with them to prevent HIV, which will only be successful if both the efficacy and effective coverage of interventions are emphasized. To be effective, interventions need to include a mechanism that directly prevents HIV transmission. These can be to reduce the risk of viral transmission through antiviral treatment or condom use, to reduce the chances of exposure through HIV testing and serosorting, or to prevent acquisition using pre‐ or post‐exposure prophylaxis [33]. In addition, for effective programmes, the environment, both legal and social, needs to be favourable for the adoption of safe behaviours, and the user‐provider interface needs to be attractive [34]. Interventions are needed to ensure that the political and human rights environment allow effective programming for key populations. In determining the cost‐effectiveness of interventions we consider the costs, which can be greatly reduced if they cover only those most at risk, and the effectiveness. Effective interventions to reach key populations might be more expensive per person reached but may also be more affordable if fewer people have to be reached. Interventions spread across the entire population may be less expensive per person reached, and better integrated into routine healthcare delivery, but if they fail to reach those most at risk or are infective among them, they will be a waste of resources.

In responding to HIV as a public health emergency much of the success has been achieved by treating HIV as being exceptional and funding HIV‐specific interventions in parallel to the existing health system. However, to maintain the HIV response efforts are being made to integrate HIV care within universal health care (UHC), reducing AIDS exceptionalism [35]. With large numbers in need of long‐term treatment such an effort makes sense, but is unlikely to meet the needs of key populations, particularly for treatment assisted management of drug use disorders, and provision of pre‐exposure prophylaxis. In moving away from AIDS exceptionalism there is a risk that key population interventions will be further neglected leading to resurgent HIV epidemics. Either the needs of key populations must be considered and included in UHC, and extra elements included in health insurance schemes, or other funding mechanisms, or needs of key populations with respect to HIV need to be provided for in addition to UHC and HIV exceptionalism be maintained.

## 3 CONCLUSIONS

As HIV incidence declines across sub‐Saharan Africa, HIV spread in high‐risk key populations is likely to be maintained unless more emphasis and resources are placed on interventions for these populations. We currently lack data on the care cascade for key populations, and too few resources from the HIV response are focused on the needs of key populations. There is a need for better epidemiological data describing key populations, more support for services directed at key populations, and more effective intervention packages for key populations. If control of HIV is maintained in key populations, then more general interventions will become redundant and allow for cost savings over time. As PLHIV will need to be treated for the long term, reducing expenditure on HIV will take decades to play out. A reliance on historic patterns of HIV acquisition and a focus on acquisition (rather than transmission) provides only a partial picture of where resources can be used most cost‐effectively to control the future spread of HIV, and is currently over‐emphasizing the importance of some low‐risk groups compared to key populations.

## Competing interests

GPG is an employee of the Bill and Melinda Gates Foundation.

## Authors’ contributions

GPG wrote this commentary.
